# A Novel Hybrid Machine Learning-Based System Using Deep Learning Techniques and Meta-Heuristic Algorithms for Various Medical Datatypes Classification

**DOI:** 10.3390/diagnostics14141469

**Published:** 2024-07-09

**Authors:** Yezi Ali Kadhim, Mehmet Serdar Guzel, Alok Mishra

**Affiliations:** 1 College of Engineering, University of Baghdad, Jadriyah, Baghdad 10071, Iraq; eng.yeziali@yahoo.com; 2 Department of Modeling and Design of Engineering Systems (MODES), Atilim University, Ankara 06830, Turkey; 3 Department of Electrical and Electronics Engineering, Atilim University, Incek, Ankara 06830, Turkey; 4 Department of Computer Engineering, Ankara University, Yenimahalle, Ankara 06100, Turkey; mehmet.serdar.guzel@ankara.edu.tr; 5 Faculty of Engineering, Norwegian University of Science and Technology, 7034 Trondheim, Norway; 6 Department of Software Engineering, Atilim University, Incek, Ankara 06830, Turkey

**Keywords:** deep learning, autoencoder, classification, medical dataset, COVID-19, brain tumor, meta-heuristic algorithm

## Abstract

Medicine is one of the fields where the advancement of computer science is making significant progress. Some diseases require an immediate diagnosis in order to improve patient outcomes. The usage of computers in medicine improves precision and accelerates data processing and diagnosis. In order to categorize biological images, hybrid machine learning, a combination of various deep learning approaches, was utilized, and a meta-heuristic algorithm was provided in this research. In addition, two different medical datasets were introduced, one covering the magnetic resonance imaging (MRI) of brain tumors and the other dealing with chest X-rays (CXRs) of COVID-19. These datasets were introduced to the combination network that contained deep learning techniques, which were based on a convolutional neural network (CNN) or autoencoder, to extract features and combine them with the next step of the meta-heuristic algorithm in order to select optimal features using the particle swarm optimization (PSO) algorithm. This combination sought to reduce the dimensionality of the datasets while maintaining the original performance of the data. This is considered an innovative method and ensures highly accurate classification results across various medical datasets. Several classifiers were employed to predict the diseases. The COVID-19 dataset found that the highest accuracy was 99.76% using the combination of CNN-PSO-SVM. In comparison, the brain tumor dataset obtained 99.51% accuracy, the highest accuracy derived using the combination method of autoencoder-PSO-KNN.

## 1. Introduction

Medicine, the cornerstone of healthcare, is pivotal in improving and sustaining human health. It encompasses a vast array of disciplines, from preventive care to sophisticated treatment modalities, all of which are aimed at enhancing the quality of life. In recent times, the focus on medicine combined with computer science has intensified in order to save time and effort and delve deeper into understanding disease and diagnosing it.

This research proposes an automated approach that leverages advantageous features to reduce prediction errors and enhance diagnostic quality. The method utilizes a variety of machine learning and deep learning techniques to classify COVID-19 and brain tumors based on the analysis of MRI and CXR images. A brain tumor is an abnormal and unwanted growth of tissue cells in the brain that causes neurological complications in patients. Nowadays, as a result of environmental and human health behaviors, instances of these tumor are quickly increasing [1]. We will need a combination of a computer-aided diagnostic (CAD) system and a medical image processing method that generates high-quality images of the afflicted bodily component, typically human soft tissues, to handle this situation. Magnetic resonance imaging is a brain imaging technique that provides significant information to allows a physician or CAD to identify whether a patient has a tumor or not and, if a tumor is identified, to discriminate between its forms so that the patient can receive suitable early treatment. Unlike X-ray imaging, MRI reveals every essential detail without exposing the patient to radiation [2]. It is a versatile method since the contrast between one tissue and another can be altered by changing the imaging method. It is possible to make images with great contrast by adjusting the radio frequency and gradient pulse, for example. There are two types of brain tumors: benign and malignant [3]. Non-cancerous tumors are benign, and cancerous tumors, called malignant tumors, are more likely to develop as a result of cancer in any region of the body, not just the brain. On the other hand, the COVID-19 pandemic, which affected the entire world in the last few months of 2019, started in China. The virus was officially named SARS-CoV-2 (severe acute respiratory syndrome-coronavirus-2) by the World Health Organization (WHO) [4]. The virus spread quickly, infecting an enormous amount of people, and the WHO declared it a “pandemic.” It is commonly known that the two main ways in which the virus spreads are by air and physical touch. This virus reportedly attacks the lungs directly, resulting in severe pneumonia [5]. Being an RNA virus, it is difficult and time-consuming to identify. Early identification is essential to reducing COVID-19’s effects [6,7]. Consequently, individuals infected with the virus have an increased chance of surviving the potentially fatal situation and receiving timely medical attention. However, this process is expensive, time-consuming, and inconvenient. Moreover, the actual sensitivity of detection is low [8,9]. This test is insufficient on its own; medical image processing methods like chest X-rays (CXR) are required to support it.

Kennedy and Eberhart developed the particle swarm optimization (PSO) technique in the middle of the 1990s. Using a huge quantity of integrated knowledge about the design space, the PSO technique publishes a set of randomly chosen solutions (the primary group) in the design space in order to find the best solution among several iterations (movements). All group members gain from this. The PSO approach is based on how flocks of birds, fish, and other animals can use information sharing to adapt to their environment, find abundant food supplies, and avoid predators like fishermen. This strategy has an evolutionary advantage. References [10,11] serve as a motion simulator for the novel optimization approach and provide a detailed history of the PSO algorithm’s development. Due to its ability to cope with continuous and discrete variables, restricted nonlinear constraints, and targets without the need for gradient information, the genetic algorithm (GA) technique is utilized to handle complicated optimization issues. This study uses the formal hypothesis testing method to examine the computational efficacy and efficiency of GA and PSO. The goal is to support the claim that, although PSO’s computing efficiency is superior to GA’s, it is just as successful at locating optimal total solutions. The outcomes of this test will be crucial for the continued development of PSO. The other sections of this article are organized as follows. Prior to formulating the hypothesis testing procedure, the PSO and GA versions employed in the comparison study are summarized. Second, the effectiveness of GA and PSO were compared using three well-known criteria. Third, two optimization issues involving the two-space systems are brought forward in order to assess how well both techniques work in real-world situations. The last two sections also present the results of this study.

The activity of ants is one of the intriguing natural behaviors for locating food, displaying a highly cognitive nature. Ants have a cunning manner of finding food that they can employ to reduce output error and discover the quickest path to food. This study first introduces the behavior of ants before introducing modified ant colony optimization for the feature selection approach. The COVID-19 and brain tumor data are classified using deep learning and autoencoder-based techniques in the second phase [12].

The primary objective of this study is to utilize PSO in conjunction with deep learning techniques to attain high accuracy in classifying COVID-19 or brain tumor datasets.

The aim of incorporating the PSO algorithm is to select significant and effective features while eliminating redundant and irrelevant ones from complex datasets inspired by the natural swarm behavior observed in fish and birds. This algorithm is integrated with deep learning techniques to extract and select optimal features, thereby improving model performance. In recent years, there has been a surge in deep learning-based studies focused on classifying brain tumors and COVID-19. For example, ref. [13] achieved an accuracy of 97.5% in MRI brain tumor classification using a CNN-based approach, simplifying the system’s complexity by employing a deeper architecture. To reduce feature dimensionality, Jaeyong et al. [14] employed an ensemble of deep feature methods. These were complemented by the support vector machine (SVM) with radial basis function (RBF) for accurate tumor classification. A hybrid feature extraction technique based on the regularized extreme learning machine was presented by Abdu Gumaei et al. [15]. They evaluated their suggested strategy using brain MRI scans. Min–max was employed for the preprocessing and augmentation of the contrast. In their study, the experiments yielded a result of 94.233%.

However, these studies have several drawbacks in terms of the complexity of implementation, parameter selection in preprocessing steps, determining the coarse structures of deep neural networks (DNNs) accurately, and the difficulty of implementing complex DNN structures and training algorithms. Furthermore, in terms of data limitations, a common challenge is the lack of an adequate amount of reliable data, and these studies are specified as having one medical dataset for training algorithms. This can lead to imbalances within the datasets used for multiclass classification. These gaps highlight the need for improved algorithms that can handle complex data. This research is motivated by the increasing complexity of diagnosing clinical big data in the context of a growing patient population, and the PSO was selected for its robust search capabilities in identifying optimal features from the data.

The principal contributions of this study are as follows:

- Development of interpretable models: We focus on developing models that not only provide accurate predictions but also offer insights into features by extracting features from different medical image datasets using several deep learning techniques; autoencoders; and pre-trained CNNs (namely, AlexNet, GoogLeNet, ResNet50, and DenseNet201). These are coupled with the next step of the PSO meta-heuristic algorithm, and the last step is that the results are predicted by a different classifier (SVM, KNN, DT, etc.). Ultimately, we utilize the particle swarm optimization (PSO) method to enhance detection precision by selecting the most significant and effective features while eliminating redundant ones from different datasets.
- The results of this study were proven through the use of two different medical datasets, the first being for a brain tumor imaged with MRI, and the second being a completely different dataset for the lungs of COVID-19 patients that were imaged with CXR.
- We demonstrate the novelty and superiority of this proposed feature selection combination over existing diagnostic baseline models.
- We validate the effectiveness of the PSO algorithm in feature selection compared to genetic algorithms (GAs) and ant colony optimization (ACO) for various medical datasets, including MRI and X-ray images. This algorithm showed superiority over other heuristic algorithms in different datasets.
- This proposed method will aid in improving understanding and interpretation by medical professionals.

The remainder of this paper is organized as follows: the related research is presented in Section 2; the datasets and their preprocessing, methodologies, deep learning techniques, and the proposed feature selector are described in Section 3; and the results are also presented, discussed, and analyzed in Section 4. Then, finally, the conclusion, limitations, and prospects for future work recommendations are given in Section 5.

## 2. Related Works

In [16], the PSO method was used for feature selection in the automated classification of brain images using wavelet energy and biogeography-based optimization. Different classifiers were used and tested; the best result was obtained using the PSO method. In [17], fusion-based feature extraction was used for COVID-19 diagnosis, and the deep learning method was used to classify the healthy and non-healthy images. Domingos Alves et al. [18] suggested using the PSO with optimized XGBoost and convolutional neural network for the classification of COVID-19 patients based on chest X-ray images. The hybrid PSO and SVM classifier model is presented in [19] for brain tumor classification. For feature learning, a brand-new deep learning model termed MRSDAE is put forth in [20]. The authors’ approach works incredibly well for extracting characteristics from vibration signals. They simultaneously developed the parameters for the proposed approach and structure using the PSO.

In [21], the authors used the PSO for feature selection from COVID-19 data and used the voting classifier algorithms on CT images. In [22], the real-time application based on PSO and one-dimensional CNN and SVM is used for the classification of medical data.

For feature extraction from MRI brain images, Kaplan et al. presented an approach based on the modified local binary pattern [23]. In their approach, the image is first normalized and smoothed using filtering, after which the two separate modified LBP methods—nLBP and LBP—are utilized. The nLBP method was shown to have a high accuracy of 94.56%. They employed machine learning techniques like ANN, Random Forest, K-NN, and decision trees for classification.

In [24], the authors propose the classification of liver and brain tumor diseases using the CNN, discrete wavelet transform, and LSTM. The dataset from Firat University, which includes 56 benign and 56 cancerous photos, is used in their research. The authors were able to accurately diagnose the liver tumor and brain tumor with 98.60% and 99.10% accuracy, respectively.

For MRI classification, Swati et al. [25] employed transfer learning and fine tuning. They applied the pre-trained CNN model and the novel transfer-learning-based fine-tuning scenario. Analyzing the 5-fold cross-validation number, they obtained 94.82% accuracy.

Brain tumor diagnosis in MRI images is conducted using a hybrid technique based on neural autoregressive distribution estimates and convolutional neural networks [26].

In [27], the authors used VGG16 and VGG19 for feature extraction. For the validation of the results, the BraTS datasets are used. The accuracy obtained from their method was 97.8%, 96.9%, and 92.5% for BraTs2015, BraTs2017, and BraTs2018, respectively.

In [28], the authors extracted features using the discrete wavelet transform (DWT) method. Pathak et al. [29] used the cost-sensitive top-2 smooth loss function to utilize and enhance the accuracy of the results.

The Bag of Words (BoW) technique was utilized by Cheng et al. [30] to extract characteristics from the photos, and SVM was used to classify the images. To identify text sources, BoW was utilized [31,32]. This approach to identifying the features of medical images is ineffective, and in [30,33,34] the authors employed deep learning with the SVM classifier as their sole feature selector in order to choose the most useful features and produce high-quality results. To extract as many of the most useful features as possible and categorize the dataset using an SVM classifier, a modified deep CNN network was employed [35].

The authors in [36] employ the 2D discrete wavelet transform (DWT) and 2D Gabor filter. The facial recognition systems that were employed in [37,38] also benefit from these feature extraction techniques. To detect and categorize images of brain cancers, Ghassemi et al. [39] coupled the Generative Adversarial Network (GAN) and ConvNet (random split). GAN techniques have recently been applied to face recognition systems [40,41]. Human faces have much higher resolutions than tumor photos, and they also have more visible objects. ConvNet, however, can be combined to achieve great accuracy.

The accuracy score of Umut Özkaya et al. [42] was 98.27% thanks to the usage of CNN and data fusion. Because data fusion produces strong features, this method can be recognized as having a higher performance compared to other methods. When the feature extraction approach used in COVID-19 pictures is not joined by a feature selection method like ANN [43], DNN [44], Random Forest [45], Tailored CNN [46], DenseNet [47], DarkNet-19-based CNN [48], or deep learning [8], we cannot suppose that the result will be of high accuracy.

In [49], S. Asif et al. significantly improved Mpox detection accuracy by using a novel CGO-Ensemble framework, which integrated transfer-learning models with feature layers and residual blocks. For weight allocation, they also the Chaos Game Optimization (CGO) algorithm on two widely recognized benchmark datasets: the Mpox Skin Lesion Dataset (MSLD) and the Mpox Skin Image Dataset (MSID). The results were 100% for MSLD and 94.16% for MSID. In [50], S. Asif et al. developed the MO-WAE model, which uses particle swarm optimization for optimal weight distribution and combines DenseNet201, MobileNet, and DenseNet169 with extra layers for better classification. This model successfully detects Mpox with an impressive 97.78% accuracy.

In [51], S. Asif et al. proposed a novel deep-stacked ensemble model called “BMRI-NET”. This model detects brain tumors from MRI results and achieves an accuracy of 98.69% on a Figshare brain MRI dataset, containing three types of brain tumors (meningiomas, gliomas, and pituitary tumors) and consisting of 3064 images. In [52], W. Wang et al. introduced a self-tuning convolutional neural network (PSTCNN) guided by PSO. Used on a dataset for COVID-19 diagnosis, this method achieved an accuracy of 93.99%, sensitivity of 93.65%, and specificity of 94.32%. In [53], S. Punitha et al. introduced a technique utilizing an Artificial Bee Colony (ABC)-optimized ANN to classify the patients into two classes, either COVID-19 or non-COVID-19. The ABC algorithm is used to optimize the ANN’s input features, initial weights, and hidden nodes. This study has an acceptable accuracy of 92.37%.

In [54], S. K. Rajeev et al. proposed a method that employed an Improved Gabor Wavelet Transform (IGWT) for feature extraction. The optimal features were selected using the Black Widow Adaptive Red Deer optimization (BWARD) algorithm, and an Elman-BiLSTM network was used to classify brain tumors from MRI images. This method achieved an accuracy of 98.4%. In [55], S. Rajakumar et al. developed a deep learning framework that was improved by a new political exponential Deer Hunting Optimization Algorithm (DHOA) and they used classifiers from the Pyramid Scene Parsing Network (PSPNet), Shepard convolutional neural network (ShCNN), and Deep CNN. This approach segments and classifies MRI images of brain tumors, achieving a 92.9% tumor classification accuracy.

In [56], Geetha et al. developed a deep learning model for the classification of brain tumors using the Sine Cosine Archimedes Optimization Algorithm (SCAOA). MRI brain images were preprocessed and segmented, and after that, the features were extracted. This model achieved a sensitivity of 92.3%, a specificity of 92.0%, and an accuracy of 93.0%.

## 3. Material and Methods

In this study, two significant types of medical datasets were implemented: MRI scans for brain tumors and X-rays for COVID-19. The images from both datasets served as inputs for the combined detection system.

Preprocessing was applied to these datasets and introduced to our approaches. Our methods involved two combinations of deep learning approaches with meta-heuristic algorithms, specifically pre-trained CNN with PSO and autoencoders with PSO. The results of the features were introduced to several classifiers in order to evaluate our approaches.

### 3.1. Datasets

#### 3.1.1. COVID-19 Dataset

The severe acute respiratory illness is caused by a coronavirus strain known as COVID-19 (coronavirus disease 2019; SARS-CoV-2). Before spreading globally, the first cases were found in Wuhan, Hubei, China, in late December 2019 [57]. The WHO classified the current illness as a pandemic on 11 March 2020.

**Figure 1:**
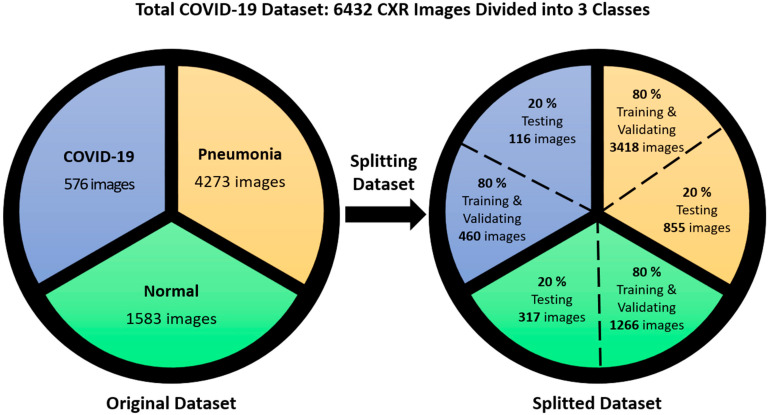
Distribution of COVID-19, pneumonia, and normal CXR images in training, validation, and testing datasets.

**Figure 2:**
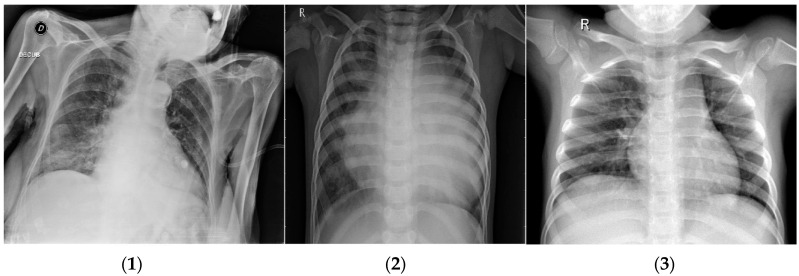
Three classes of COVID-19 dataset: (**1**) COVID-19; (**2**) pneumonia, and (**3**) normal.

The dataset contains 6432 X-ray images in total [58]; image sizes vary and are not fixed. All images have been modified. This dataset is organized so that 80% is used for the training of total images and the remainder is used for the test dataset represented in Figure 1. It consists of three classes: COVID-19, pneumonia, and normal. These images are divided into 460 COVID-19, 3418 pneumonia, and 1266 normal images for the training and validation of the model. Also, we use 116 COVID-19, 855 pneumonia, and 317 normal samples to test the model. A sample from each class in the COVID-19 dataset is shown in Figure 2.

#### 3.1.2. Brain Tumor Dataset

**Figure 3:**
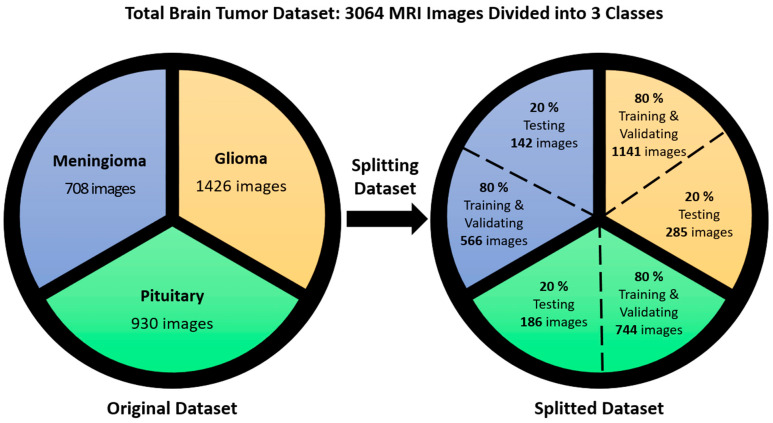
Distribution of meningioma, glioma, and pituitary MRI images in training, validation, and testing dataset.

**Figure 4:**
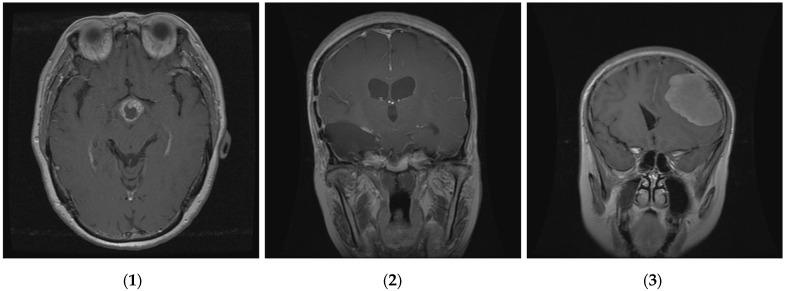
Three classes of brain tumor datasets; (**1**) meningioma, (**2**) glioma, and (**3**) pituitary brain tumors.

The proposed method was examined and tested by using data on brain tumors gathered between 2005 and 2010 from Tianjin Medical University General Hospital and Nanfang Hospital in Guangzhou, Guangdong, China [59]. Three distinct types of brain tumors are represented in this brain tumor dataset, which consists of 3064 T1-weighted contrast-enhanced pictures from 233 patients: meningioma (708 slices), glioma (1426 slices), and pituitary tumor (930 slices). We randomly divided these images into two groups, using 80% for training and validation and 20% for testing the model. This means that the pituitary sample is divided into 744 slices to train the model and 186 slices for the test; the glioma sample is divided into 1141 slices for training and the remaining (285 slices) are used for the test; and the meningioma is divided into 566 slices for training the model and 142 slices for testing. The results are represented in Figure 3. The images are 512 × 512 pixels in size and are available as .png files. Figure 4 presents sample images from the brain tumor dataset, showcasing examples from each of the three tumor classes.

#### 3.1.3. Dataset Preprocessing

**Figure 5:**
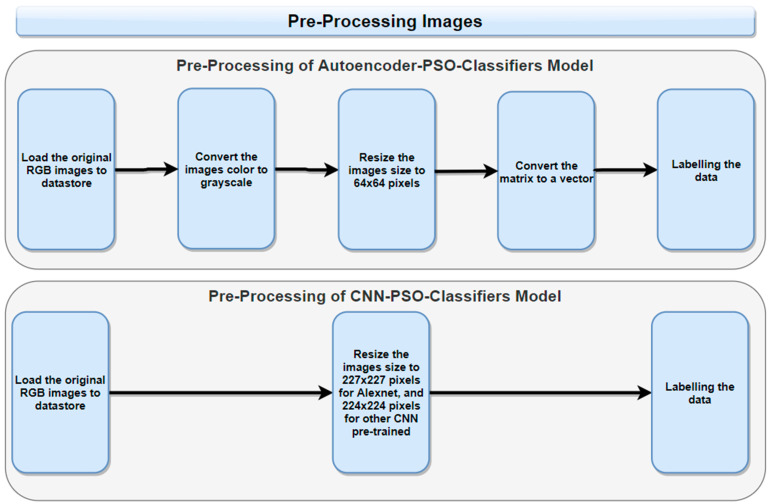
Image dataset preprocessing steps.

Each brain tumor image is provided at a resolution of 512 × 512 pixels, while COVID-19 images vary in size. It is important to prepare these image datasets beforehand to improve the quality of features, which helps to enhance predictions for both CXR and MRI images. The processed data are then used for feature extraction using either an autoencoder with PSO or a CNN with a PSO model, as shown in Figure 5.

Two different preprocesses are used for each model. For the autoencoder with the PSO model, the original RGB images are changed to grayscale. They are resized to be 64 × 64 pixels. The final preprocessing step includes converting these images (matrices) into arrays (vectors) before feeding them into the model. On the other hand, for the model of CNN with PSO, preprocessing involves resizing the images to 227 × 227 pixels for Alexnet, and 224 × 224 for the other CNN pre-trained model used.

### 3.2. Methodology

**Figure 6:**
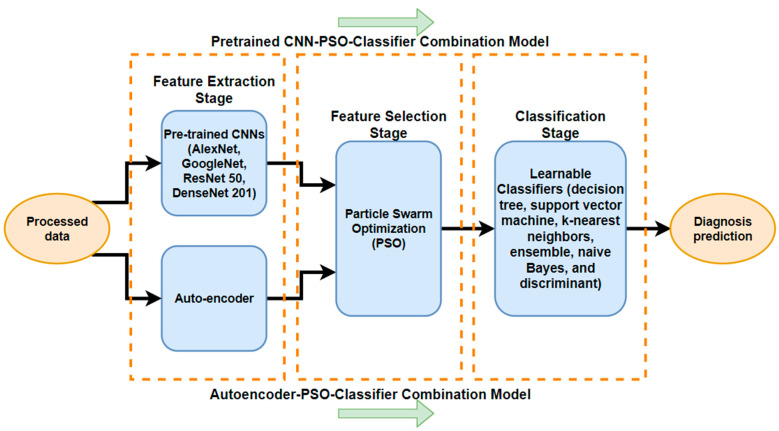
The diagram for different combinations of the proposed models.

In the training stage for the first combined model, we applied these training modified and augmented images to the autoencoder network in order to perform feature extraction. After that, the aid of the PSO feature selection algorithm enhanced the accuracy of our model in choosing the best features. After obtaining the best features, we applied several learnable classifiers such as discriminant, ensemble, Naive Bayes, support vector machine, decision tree, and k-nearest neighbors. These classifiers categorize data based on the labels of the input type and learn from the characteristics extracted via the PSO method. To present the quality of this study for the diagnosis of the disease, we applied the PSO feature selection algorithm with other deep learning techniques. We present a second combination that can deal in particular with image processing. The convolutional neural network is a deep learning method. We applied several pre-trained CNNs to train the model and extract features from the input dataset. Furthermore, PSO was implemented to select features. After that, the selected features were introduced into learnable classifiers for the detection of our problems. To prove the results, the outcomes of evaluating several parameters were calculated, such as accuracy, sensitivity, specificity, etc. The classifiers were trained in a supervised fashion to learn the weights of each label and tested to calculate the learning rate. Figure 6 shows the overall framework of the proposed system.

#### 3.2.1. Feature Extraction with CNN

**Figure 7:**
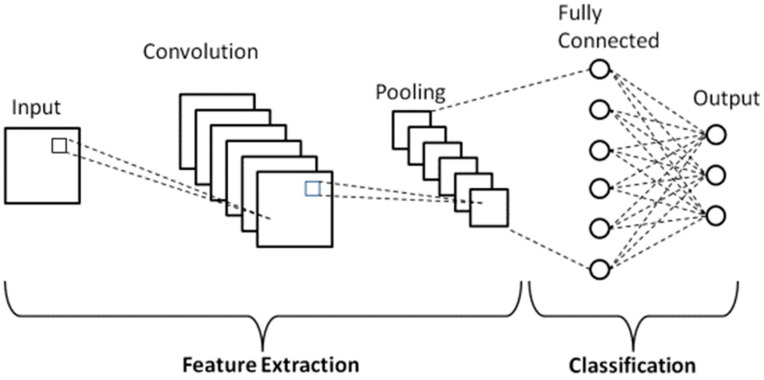
Basic CNN structure.

The pre-trained convolutional neural network model is used in the initial stage to extract significant features from the image. The pre-trained technique is one of the pre-trained procedures that are used. A popular model, used in many studies on picture categorization, is AlexNet. This step aims to extract sensitive and high-level information from input photos. This model embodies the science of human eyesight. Input, convolution, pooling, and fully connected layers are among the layers that make up CNNs, as shown in Figure 7, which represents a simple CNN structure. The core operations of a convolutional neural network model are contained in this structure. As a result of its use of the shared weight technique rather than the fully connected technique to reduce computation, CNN is now frequently used to solve a variety of computer vision problems. From one model to another, the following different features are extracted:

- Alexnet: Input size is 227 × 227 × 3, and the number of features is 4096.
- Googlenet: Input size is 224 × 224 × 3, and the number of features is 1000.
- Resnet50: Input size is 224 × 224 × 3, and the number of features is 2048.
- Densenet201: Input size is 224 × 224 × 3, and the number of features is 1920.

#### 3.2.2. Feature Extraction with Autoencoders

An autoencoder is a neural network that has been trained to replicate its input at its output. Deep neural networks can be trained using autoencoders. Insofar as no labeled data are required, the process of training an autoencoder is unsupervised. The optimization of a cost function continues to be the foundation of the training process. The discrepancy between the input *x* and the output reconstruction $\hat{x}$ is measured by the cost function.

An encoder plus a decoder comprises an autoencoder. Although the encoder and decoder can each have numerous levels, let us assume for the sake of simplicity that they each only have one layer.

When an autoencoder receives a vector as input, $x\in R^{D_{x}}$, it maps that vector onto another vector, $z\in R^{D^{\left(1\right)}}$, as seen below:

(1) $$z=h^{\left(1\right)}(W^{\left(1\right)}x+b^{\left(1\right)})$$

where the first layer is denoted by the superscript (1). The encoder’s transfer function is represented by $h^{\left(1\right)}:R^{D^{\left(1\right)}}\to R^{D^{\left(1\right)}}$, its weight matrix is represented by $W^{\left(1\right)}\in R^{D^{\left(1\right)}\times D_{x}}$, and its bias vector is represented by $b^{\left(1\right)}\in R^{D^{\left(1\right)}}$. The decoder then approximates the original input vector, *x*, from the encoded representation, *z*, as follows:

(2) $$\hat{x}=h^{\left(2\right)}(W^{\left(2\right)}z+b^{2})$$

where the second layer is indicated by the superscript (2), the decoder’s transfer function is represented by $h^{\left(2\right)}:R^{D_{x}}\to R^{D_{x}}$, its weight matrix is represented by $W^{\left(1\right)}\in R^{D_{x}\times D^{\left(1\right)}}$, and its bias vector is represented by $b^{\left(2\right)}\in R^{D_{x}}$.

##### Sparse Autoencoders

By including a regularizer in the cost function, it is possible to promote the sparsity of an autoencoder [60,61]. This regularizer is based on a neuron’s average output activation value. A neuron’s *i* average output activity measure is calcualted as follows:

(3) $$\hat{\rho_{i}}=\frac{1}{n}\sum_{j=1}^{n}z_{i}^{\left(1\right)}\left(x_{j}\right)=\frac{1}{n}\sum_{j=1}^{n}h(w_{i}^{\left(1\right)T}x_{j}+b_{i}^{\left(1\right)})$$

where *n* represents the overall quantity of training samples, the jth training sample is *x_j_*, $b^{\left(1\right)}$ is the ith entry of the bias vector of $b_{i}^{\left(1\right)}$, and $w_{i}^{\left(1\right)T}$ is the ith row of the weight matrix $W^{\left(1\right)}$. A neuron is said to be “firing” if its output activation value is high. Only a few of the training instances activate the hidden layer neuron, as shown by its low output activation value. By adding a term to the cost function in a way that limits the values of $\hat{\rho_{i}}$, keeping them low, the autoencoder is compelled to learn a representation where each hidden layer neuron fires in a constrained number of training samples. In other words, by reacting to a trait that appears in a very small percentage of the training instances, each neuron carves out a niche for itself.

##### Sparsity Regularization

The sparsity regularizer makes an effort to restrict the output of the sparsity of the buried layer. When a neuron’s average activation value, $\hat{\rho_{i}}$, and its target value, I, do not have a similar value, it is possible to promote sparsity by using a regularization term that takes a big value [61,62]. Kullback–Leibler divergence is one possible name for sparsity regularization.

(4) $$\Omega_{sparsity}=\sum_{i=1}^{D^{\left(1\right)}}KL(\rho ∥\hat{\rho_{i}})=\sum_{i=1}^{D^{\left(1\right)}}\rho \log\left(\frac{\rho }{\hat{\rho_{i}}}\right)+(1-\rho )\log(\frac{1-\rho }{1-\hat{\rho_{i}}})$$

Kullback–Leibler divergence is a function that assesses the degree of separation between two distributions. In this instance, it has a value of zero when a value of *ρ* and $\hat{\rho_{i}}$ are equal to each other and increases as they diverge. This term must be modest in order to minimize the cost function; as a result, *ρ* and $\hat{\rho_{i}}$ must be relatively close to one another. When training an autoencoder, the Sparsity Proportion name–value pair input allows you to specify the desired average activation value.

##### *L*_2_ Regularization

The sparsity regularizer can be made to have minimum values by increasing the weights *w*^(*l*)^ and decreasing the values of *z*^(1)^ while training a sparse autoencoder [60,61]. By applying a regularization term to the weights in the cost function. This term penalizes large weights by adding their squared values to the cost functions. The so-called *L*_2_ regularization term is defined as follows:

(5) $$\Omega_{weights}=\frac{1}{2}\sum_{l}^{L}\sum_{j}^{n}\sum_{i}^{k}{\left(w_{ji}^{\left(l\right)}\right)}^{2}$$

*L* is the number of covert layers.

There are two options for the transfer function: logistic sigmoid function and positive saturating linear transfer function. These transfer functions are illustrated in Equations (6) and (7), respectively.

(6) $$f\left(x\right)=\frac{1}{1+e^{-x}}$$

(7) $$f\left(x\right)=\left\{\begin{array}{cc} 0, & if\;x\leq 0\\ x, & if\;0<x<1\\ 1, & if\;x\geq 1 \end{array}\right.$$

**Figure 8:**
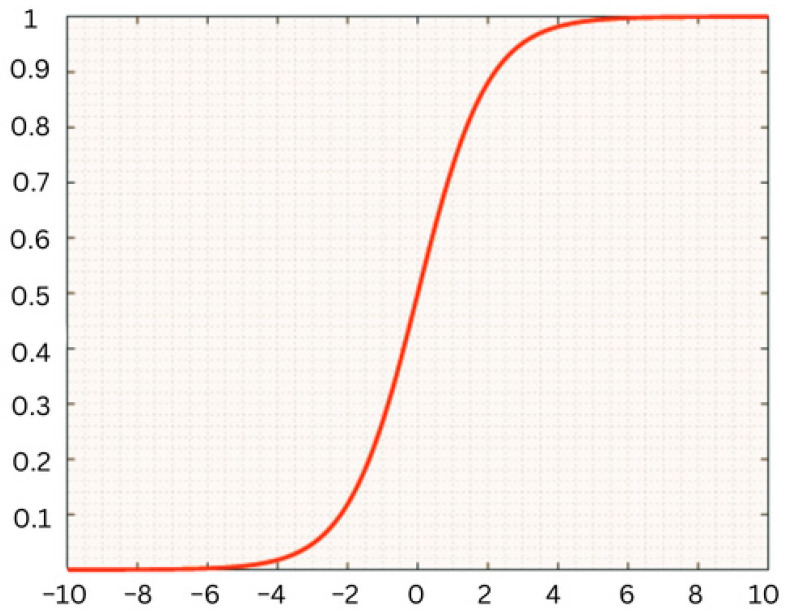
Sigmoid function.

For the encoder transfer function, we used the sigmoid function. This function’s curve is shown in Figure 8.

##### Deep Sparse Autoencoders

The comma-separated pair consisting of the loss function specifies the cost function to utilize for training and mean square sparsity. The cost function for training a sparse autoencoder is the adjusted mean squared error function [60]:

(8) $$E=\underset{Mean\;squared\;error}{\underbrace{\frac{1}{N}\sum_{n=1}^{N}\sum_{k=1}^{K}{\left(x_{kn}-\hat{x_{kn}}\right)}^{2}}}+\lambda \times \underset{\begin{array}{c} L_{2}\\ regularization \end{array}}{\underbrace{\Omega_{weights}}}+\beta \times \underset{\begin{array}{c} sparsity\\ regularization \end{array}}{\underbrace{\Omega_{sparsity}}}$$

where *λ* is the sparsity regularization term’s coefficient and is the *L*_2_ regularization term’s coefficient *β*. While training an autoencoder, you can provide the values of *λ* and *β* by using the *L*_2_ weight regularization and sparsity regularization name–value pair arguments, respectively.

#### 3.2.3. Particle Swarm Optimization Algorithm for Feature Selection

##### PSO Explanation and Literature

To overcome the issue of interpreting pictures in computer animations that attempt to mimic natural events, the hypothesis of employing a multitude of agents (particles, populations) that interact with simple natural techniques to construct seemingly intricate disability behaviors was adopted. As part of his research at Lacasse Film, Rios, one of the field’s pioneers, used particle devices, which individually have several components that combine to generate a fuzzy function. A succession of moving points that were normally started at predetermined positions was generated at random by the particle machine. Color, texture, a finite lifespan, and other aspects were included in the graphic simulation. Multiple random factors were used to alter the velocity vectors. Then, by adopting the velocity vector, each particle moved on to the following position, disengaging from its previous location. A forced angle was used to alter the location in order to make the move appear natural. In-depth research was performed on these systems to produce social results and real-world interactions in graphical settings. For some animations (such as a bird population), it was important to portray group behaviors with higher levels of dynamism than mere particles. It was conceivable to create a file that started member activity, but it was quite laborious, and it was also challenging to come up with a response that sounded natural. Reynolds’ higher-level group algorithm was built on the Rios particle system. He took into account the particle’s prior motion and added additional elements to it including inclinations, positional identification, and information correspondence. The group members’ extra actions were in accordance with the fundamental principles of group membership, such as the need to avoid collisions, adjust one’s velocity vector to that of the group as a whole, and be in a better position than the others. While enhancing member intelligence, the development of these fundamental models did away with the requirement for route registration. However, giving people more liberty might lead to issues like incompatibility. In order to tackle the issue, Reynolds made a specific move based on the importance of superiority. But once more, the choice may be arbitrary and illogical. Consider a gadget with a straightforward implementation, in which every particle is aware of the motions that are occurring throughout the entire population. In this instance, when the population particle count rises, the problem may become extremely challenging or perhaps impossible. Reynolds suggested the neighborhood system as a solution, which is also employed in nature because of members’ limited visibility, despite current studies suggesting that this strategy alters the population’s impaired behavior. By including social behaviors, Kennedy and Haharat aimed to expand Reynolds’ model. More significantly, they switched the Hepner and Greenander algorithm’s straightforward goal of finding a nest, developed from a periodic group algorithm, with the more practical one of locating food. This inspired academics to apply this approach to challenging mathematical issues. The objective function of the issue is viewed, in terms of these methods, as a function of population fitness. Because they are more universal than the bird model, they are now referred to as representational. A more effective and straightforward model was created by eliminating the redundant and inefficient variables [62,63,64,65,66].

##### PSO for Feature Selection

Obtaining the optimum solution for the entire swarm and each individual particle is the goal of PSO, which changes particle position and velocity over time. Equations that are based on the following velocity equation are used to update the particle positions and velocities iteratively and uniform random variables between 0 and 1 are used to generate the random variation. Where *v*_*i*_, *k* is the inertia factor, *α* is the self-confidence learning constant, *β* is the swarm influence learning constant, *r*_1_, and *r*_2_ are random values between zero and one, and *v_i_*, *k* is the velocity of particle *i* on iteration *k*. Particle *i* has never achieved a better position than PB, and any member of the population has never achieved a better position than GB. The particle position is *x*_*i*_, *k*.

The resulting algorithm for calculating the next particle position was expressed as follows:

(9) $$v_{t+1}=v_{t}+\varphi_{1}\beta_{1}\left(p_{i}-x_{i}\right)+\varphi_{1}\beta_{1}\left(p_{g}-x_{i}\right)$$

(10) $$v_{i,k+1}=w\times v_{i,k}+\alpha \times r_{1}*\left(PB-x_{i,k}\right)+\beta \times r_{2}\times \left(GB-x_{i,k}\right)$$

(11) $$x_{t+1}=v_{t}+v_{t+1}$$

(12) $$x_{i,k+1}=x_{i,k}+v_{i,k}$$

**Figure 9:**
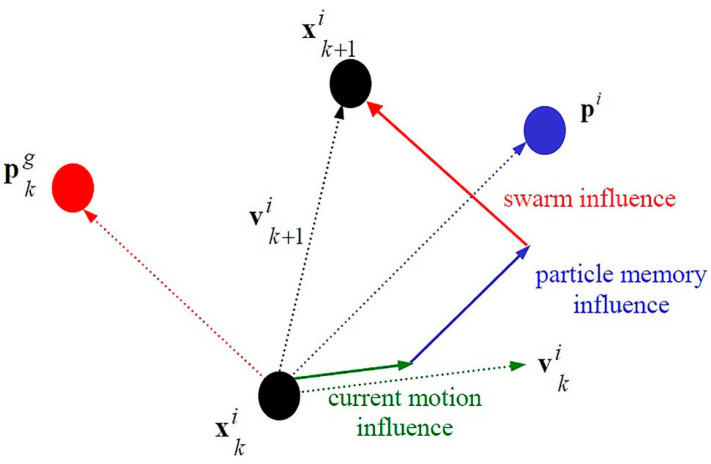
Velocity and Position situations [67].

In Figure 9, where *p_i_* is the best local solution and *p_g_* is the best global solution, it is shown how particle location and velocity are updated. Particle swarms converge to similar solutions significantly more quickly and effectively than genetic algorithms, according to a considerable amount of research conducted by Hassan et al. [67].

**Figure 10:**
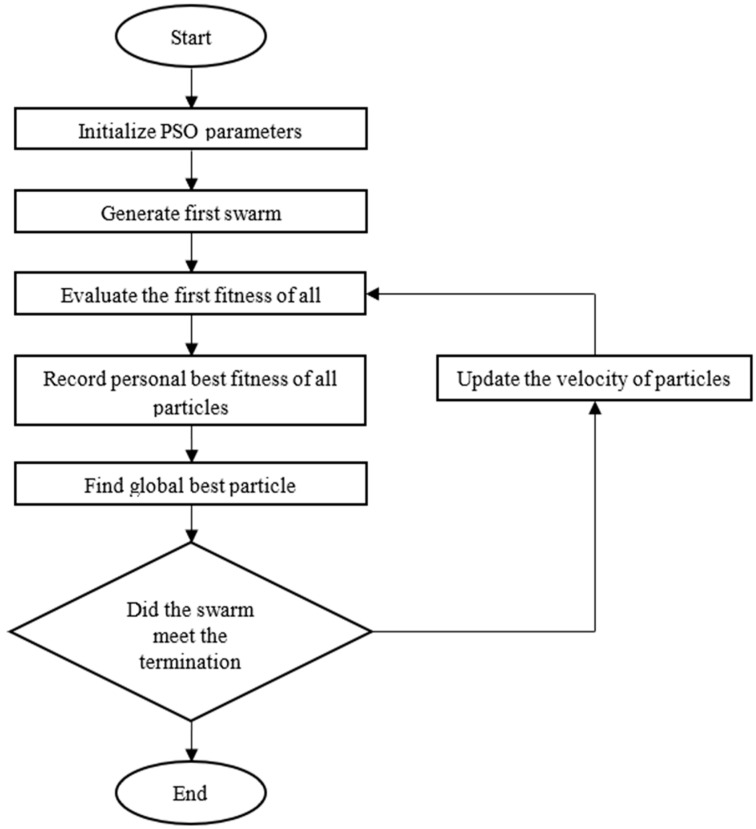
PSO flowchart.

**Figure 11:**
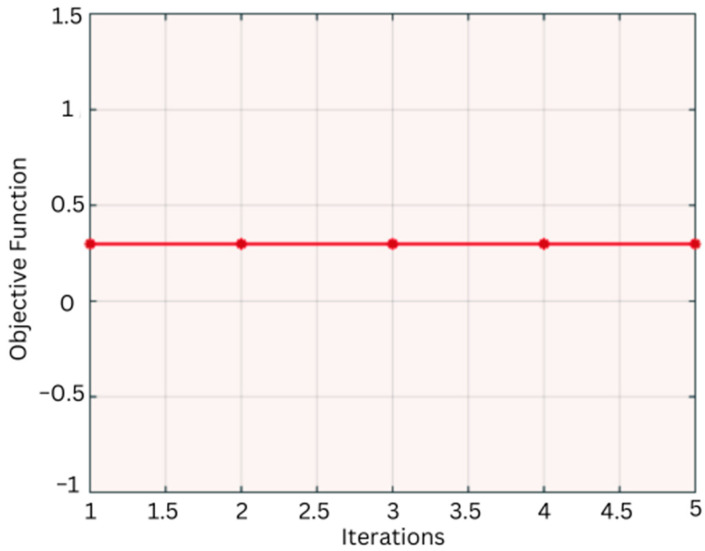
Convergence of objective function.

Figure 10 depicts the algorithm’s flowchart, which demonstrates how the algorithm works. Moreover, the objective function results in convergence, as illustrated in Figure 11.

## 4. Results and Discussion

The approach proposed in this study was trained on an Intel(R) Core(TM) i7-6500U CPU at 2.50 GHz and 2.60 GHz using MATLAB 2021b. A comprehensive study was conducted to determine the best combined technique. In the first stage, an autoencoder method was applied to two common medical datasets, featuring COVID-19 and brain tumor patients. We utilized the PSO method to separate out and choose the key features from the input training dataset. On the other hand, we applied a pre-trained CNN with the PSO algorithm to classify both of the datasets that were used in the first model. The performance of models derived from the confusion matrix was measured using several key criteria, including F1-score, recall, accuracy, precision, and others. For multiclass classification, overall accuracy, class detection rate, and class FP rate are used. True Positive (TP), True Negative (TN), False Positive (FP), and False Negative (FN), standing for positive and negative instances, respectively, are our basic terms. True Positive (TP) denotes positive instances that are classified as positive, True Negative (TN) denotes negative instances that are classified as negative, and False Positive (FP) indicates false instances that are classified as positive.

### 4.1. Autoencoder with PSO for COVID-19 Dataset

In this study, different scenarios are implemented and evaluated to ensure the performance of this model and to perform a comprehensive comparison of the combinations. In the first stage, autoencoder was applied to a COVID-19 dataset with 6 different types of classifiers. Table 1 shows the result for the autoencoder with the PSO algorithm for the COVID-19 dataset.

Accuracy is the most important parameter for evaluating this classification model, and this depends on the true values of the classified tested image, The SVM classifier obtained the higher accuracy rate which is 98.83%. Also, the misclassification rate is 1.17%.

### 4.2. Autoencoder with PSO for Brain Tumor Dataset

This model has proven successful in classifying medical images by applying these second famous data for brain tumors consisting of 3 different classes as well (meningioma, glioma, and pituitary brain tumors). Table 2 shows the result for the autoencoder with the PSO algorithm for the brain tumor dataset.

The implementation of an autoencoder training model, with the aid of a PSO algorithm, to select the best features was accurate; we obtained good quality in the classification of brain tumor classes, and the KNN classifier obtained 99.51% accuracy.

### 4.3. Pre-Trained CNN with PSO for COVID-19 Dataset

For the second scenario, several pre-trained CNNs (Alexnet, Googlenet, Resnet50, and Densenet201) were applied. CNN is a form of artificial neural network, used in image processing and detection, that was created primarily to process pixel input. A CNN uses multilayer perceptron-like technology that is designed for modest processing demands. It was also applied with PSO in order to verify the power of the feature selection method used in this study. Also, 6 types of classifiers were implemented to compute the classification model. Table 3 represents the result of four pre-trained CNNs with PSO for the COVID-19 dataset.

As stated in Table 3, the Resnet-50 pre-trained network outperformed the other three pre-trained CNNs when evaluated with the PSO feature selection algorithm. For the COVID-19 dataset, the SVM classifier produced a spectacular result with 99.76% accuracy classification.

### 4.4. Pre-Trained CNN with PSO for Brain Tumor Dataset

The brain tumor dataset was used in this evaluation of pre-trained CNN with PSO. Table 4 shows the diagnosis rates for a brain tumor in this model.

When applying the pre-trained Resnet-50, the discriminant classifier obtains a respectable 98.85% accuracy, as shown in Table 4.

### 4.5. Comparison Tables

Table 5 Shows the comparison of the previous studies performed with the proposed method for medical dataset diagnosis.

In [19], the SVM classifier was used to perform the MRI brain tumor after a machine learning model was built, using the PSO approach for feature selection.

For the two classes (benign and malignant), the hybrid model of PSO and SVM obtained 95.23%, which was an acceptable result compared to the SVM, which only obtained 86.82%, a reasonable result, indicating that the feature selection impacts on the classification process [73], while the PSO algorithm was successful in the hybrid model. There is no feature extraction method in this hybrid model. For this reason, our proposed method had the luck to obtain a higher accuracy rate, even though the proposed method was tested for a three-class dataset.

The accuracies of the approaches utilized by GLCM [68,69] are 82.00% and 96.50%, respectively. The gray-level co-occurrence matrix is helpful for textured photos like fingerprint and palmprint images because it may provide accurate findings for this type of dataset [74]. Additionally, because textural features cannot be relevant in data images, the method employed in [68], which used the GLCM, could not produce high-quality results. However, the authors combined the GLCM with the pre-trained VGG-16 CNN [69] and found that the results were improved. This indicates that employing GLCM alone will not improve a system’s performance. The VGG-19, created by the Softmax classifier, was utilized by the authors in [75] as well, and it achieved 94.58% accuracy. This problem demonstrates that the authors’ high-accuracy findings were obtained in [69] when the GLCM and VGG-19 were combined.

However, in [17], more combinations were introduced in order to achieve higher accuracy than the above studies. This study introduces FM-CNN, a fusion-based feature extraction model that uses a convolutional neural network (CNN) to perform automated COVID-19 diagnosis. Preprocessing, feature extraction, and classification are the three key aspects of the FM-CNN model. Initially, noise is removed from the input chest X-ray (CXR) pictures using preprocessing based on Wiener Filtering (WF). The preprocessed images are then sent into a fusion-based feature extraction model that uses local binary patterns, the gray-level co-occurrence matrix, and the gray-level run-length matrix (LBP). Finally, the particle swarm optimization (PSO) algorithm is used to select the best subset of characteristics. It shows that, for three classes (COVID-19, pneumonia, normal) of dataset used, the performance of this model was 98.06%, which was lower than that of the proposed method, 99.76%. As mentioned above, GLCM is effective in determining the texture of images [74]. The spatial plane features of each pixel are extracted using the texture representation model GLRM in relation to the high-order statistics [76], and the LBP is an efficient method used for texture feature extraction. This method is very popular for face detection and pattern recognition approaches [77]. Here, PSO proved its efficacy by selecting features and enhancing the accuracy of the diagnosis of the medical dataset. However, this model obtained a lower performance compared with the proposed method.

In [78], the authors performed texture feature extraction in addition to PCA to identify the best feature. The best features from medical data cannot be found via PCA feature selection [79]. Additionally, none of the other approaches listed in Table 5 apply combination methods for feature extraction and feature selection. The outcomes cannot be improved using ineffectual characteristics. Additionally, the use of comparable approaches for feature extraction and selection using the same dataset that we utilized provide the basis for this related research.

Based on the intensity and texture features that Sachdeva et al. [78] recommended, the PCA was made. The features will be more likely to locate the high Eigenvalues in the image when using this approach to PCA, which finds the Eigenvalues of the features. However, in images of brain tumors, some areas of the image are ineffective at high Eigenvalues, leading to some errors and a less-than-accurate result.

The features from pictures of brain tumors and COVID-19 are extracted using the capsule networks (CapsNets) in [50,70,80]. Two-dimensional signals, such as those employed recently in voice recognition signals and medical imaging, cannot be produced with great accuracy using this method [81]. The performance for 1D signals is superior to that OF 2D signals.

A deep convolutional autoencoder alone cannot produce satisfactory results [71,72]. More unimportant features are extracted from the images by the autoencoder. Choose the finest option by boosting these aspects.

For the proposed method, the highest accuracy of 99.76% was achieved by the model that contained CNN Resnet50 for feature extraction with the PSO algorithm for the selection of the features and the classification performed by the SVM learnable classifier, which depends on the last best features obtained from this model. PSO is a derivative-free methodology, like other heuristic optimization techniques, and is one of the most effective ways of solving global optimization problems. PSO has a straightforward concept and coding implementation when compared to other heuristic optimization methods. PSO is less sensitive to kinds of objective function than conventional mathematical techniques and other heuristic techniques [82]. There are some limitations of the PSO method [83]: it is unable to solve problems involving non-coordinated systems, such as the solution to the energy field and the moving rules of the particles in the energy field. Partially optimistic thinking is common in this method, resulting in less speed and directional control. The proposed method has been compared with another meta-heuristic method, Ant Colony Optimization (ACO), in relation to the time and accuracy of models, as shown in Table 6.

Table 6 shows that the combination model of the deep learning method with the PSO feature selection algorithm had a much longer consumption time than the same method with the ACO algorithm since the ant colonies’ aging behaviors were the inspiration for the ACO. Ants’ indirect communication lies at the heart of this behavior, allowing them to find quick routes between their nest and food sources [83].

Both the ACO and PSO algorithms are data clustering algorithms since they employ swarm dynamics. The ACO, however, works flawlessly when applied to problems with clear sources and destinations. PSO is a clustering method with numerous objectives, dynamic optimization, and constraint handling all at once. PSO is more appropriate for issues that call for ambiguous solutions, while ACO is more appropriate for issues that call for clear-cut answers [83].

Because it avoids becoming stuck in the local minimum, ant colony optimization surpasses the genetic method in speed to the global minimum point (GA). ACO aims to find the best answers to numerous optimization issues by replicating the cognitive behavior of ants. Due to its benefits, which include ease of implementation, a limited number of parameters, adaptability, etc., it has attracted significant interest on a global scale [84]. Ant colony optimization is straightforward, adaptable, reliable, scalable, and self-organizing. Compared to a genetic algorithm, it has fewer control parameters (GAs). Individuals are capable of carrying out several tasks at once. In comparison to GAs, swarm intelligence (SI) uses less memory. ACOs have a number of benefits, and many parties benefit from this type of care. The patient community gains in many different ways, such as improved outcomes, higher-quality care, more provider engagement, and a general decline in out-of-pocket costs. These techniques have an advantage over methods employing simulated annealing and genetic algorithms when the graph may alter dynamically since the ant colony algorithm can run continuously and adapt to changes in real time [84].

For this reason, the performance of the deep learning method can be much more accurate in classifying the medical dataset when it is combined with meta-heuristic algorithms for feature selection.

## 5. Conclusions

In this study, five models were employed on various medical datasets, each comprising two stages: a deep learning technique and a meta-heuristic algorithm. The models, totaling ten combinations, were evaluated with six learnable classifiers for optimal detection accuracy. The first stage involved feature extraction using either an autoencoder or pre-trained CNNs (AlexNet, GoogleNet, ResNet 50, or DenseNet 201). The second stage utilized meta-heuristic algorithms, using either PSO, ACO, or GA. PSO was used and showed its superior performance, as shown in Table 6, in terms of enhancing feature selection and improving accuracy by choosing the most noticeable visual attributes to limit the volume of data that needed to be processed across CXR and MRI datasets. Finally, the selected features were classified by the third stage using learnable classifiers decision tree, SVM, KNN, ensemble, Naive Bayes, and discriminant classifiers to process the acquired features to assess the model’s correctness.

This study achieved a satisfying classification accuracy using these combined models on CXR and MRI images of COVID-19 and brain tumor patients, respectively. The best model for the CXR dataset was ResNet-50 combined with PSO and SVM, achieving 99.76% accuracy. For the MRI dataset, the highest accuracy of 99.51% was obtained using the combination of an autoencoder, PSO, and KNN.

To lower the misclassification error rate, the PSO meta-heuristic method is used to look for the most crucial features in the accessible feature set.

The effectiveness of the suggested approach is assessed using two different medical datasets with different features, which are the CXR and MRI image datasets. The CXR information is utilized to determine the patient’s status as either COVID-19, pneumonia, and normal while using MRI images to determine the type of brain tumor present (meningioma, glioma, and pituitary). The accuracy of the entire system is indeed significantly impacted by the removal of weak, redundant, and noisy elements. When compared to the other cutting-edge techniques listed in Table 5, the suggested system has the highest accuracy performance. The main goal of the proposed accurate approach is to assist the medical field in performing earlier status detection.

## 6. Limitations

The limitations of this work include the following concerns:

1. Its reliance on supervised learning with labeled data limits automation and potential inability to fully exploit deep learning’s potential.
2. The constraint of a small dataset in the medical field restricts the generalizability and robustness of an approach.
3. The need for human participation in the diagnostic prediction process hinders scalability and real-time application in clinical settings.
4. The deep learning models employed are computationally intensive, requiring substantial hardware resources for training and inference, which may limit their applicability in resource-constrained settings.
5. The complexity of segmenting brain tumors and COVID-19 lesions, which may require specialized expertise and resources.

## 7. Future Work

1. We should aim to include a larger and more diverse dataset, covering various demographics and imaging devices, to improve the robustness and generalizability of the models.
2. We should address data labeling challenges, as the process can be time-consuming and error-prone, affecting model quality.
3. Involving close collaboration with healthcare professionals to ensure the method’s relevance, feasibility, and usability in clinical practice can lead to locating the exact segmentation of the tumor.
4. After the classification of these tumors, the segmentation of tumors is needed in order to perform exact detection: knowing the shape and size will aid the doctors in identifying the level at which the tumor is.
5. Focus on optimizing model architectures and hyperparameters to enhance performance while reducing computational requirements, making the models more feasible for real-time clinical use.

## Figures and Tables

**Table 1:** Autoencoder with PSO feature selection method on COVID-19 dataset.

Method	ACC	TPR	TNR	FPR	FNR	PPV	NPV	F1-Score	MR
Decision Tree	98.05	93.33	98.47	1.53	6.67	84.48	99.40	88.68	1.95
SVM	**98.83**	97.19	98.98	1.02	2.81	89.65	99.74	93.27	1.17
KNN	97.90	92.38	98.39	1.61	7.62	83.62	99.31	87.78	2.10
Ensemble	98.29	94.33	98.64	1.36	5.67	86.2	99.48	90.08	1.71
Naïve Bayes	90.37	87.23	99.34	0.66	12.77	93.96	90.01	90.47	9.63
Discriminant	98.60	97.11	98.73	1.27	2.89	87.06	99.74	91.81	1.40

**Table 2:** Autoencoder with PSO feature selection method on brain tumor dataset.

Method	ACC	TPR	TNR	FPR	FNR	PPV	NPV	F1-Score	MR
Decision Tree	94.12	89.47	96.21	3.79	10.53	91.39	95.31	90.42	5.88
SVM	95.75	89.6	98.78	1.22	10.4	97.31	95.08	93.30	4.25
KNN	**99.51**	98.41	99.94	0.06	1.59	99.98	99.29	99.19	0.49
Ensemble	94.61	87.68	98.04	1.96	12.32	95.69	94.14	91.51	5.39
Naïve Bayes	79.44	76.93	98.39	1.61	23.07	97.31	71.66	85.93	20.56
Discriminant	97.22	92.46	99.51	0.49	7.54	98.92	96.48	95.58	2.78

**Table 3:** Multi-pre-trained CNN using the COVID-19 dataset’s PSO feature selection approach.

Pre-Trained CNN with PSO (COVID-19 Dataset)									
Classifiers	ACC	TPR	TNR	FPR	FNR	PPV	NPV	F1-SCORE	MR
**Pre-trained CNN (AlexNet) + PSO**									
Decision Tree	96.42	89.74	97.63	2.37	10.26	90.05	98.46	89.89	3.58
SVM	99.45	99.92	99.40	0.60	0.08	96.95	99.11	98.41	0.55
KNN	98.52	99.10	98.40	1.60	0.90	93.33	98.4	96.13	1.48
Ensemble	98.91	98.74	98.81	1.19	1.26	97.42	98.52	98.08	1.09
Naïve Bayes	95.72	96.36	99.46	0.54	3.64	94.82	95.81	95.58	4.28
Discriminant	99.68	99.87	99.65	0.35	0.13	97.89	99.14	98.87	0.32
**Pre-trained CNN (GoogleNet) + PSO**									
Decision Tree	96.97	90.13	98.20	1.80	9.87	92.16	98.89	91.13	3.03
SVM	98.75	99.01	98.90	1.10	0.99	96.02	99.91	97.49	1.25
KNN	98.83	99.08	98.73	1.27	0.92	92.98	99.91	95.93	1.17
Ensemble	98.75	99.01	98.73	1.27	0.99	96.60	99.91	97.79	1.25
Naïve Bayes	98.52	96.40	99.23	0.77	3.60	93.05	99.14	94.70	1.48
Discriminant	98.83	99.02	98.81	1.19	0.98	97.19	99.91	98.10	1.17
**Pre-trained CNN (ResNet 50) + PSO**									
Decision Tree	97.51	92.85	97.89	2.11	7.15	90.29	99.40	91.55	2.49
SVM	**99.76**	99.89	99.74	0.26	0.11	97.41	99.18	98.63	0.24
KNN	98.99	98.13	99.06	0.94	1.87	94.38	99.82	96.22	1.01
Ensemble	99.22	99.81	99.15	0.85	0.19	96.49	99.60	98.12	0.78
Naïve Bayes	99.06	98.82	99.91	0.09	1.18	99.13	99.06	98.97	0.94
Discriminant	99.66	99.40	99.64	0.36	0.60	97.31	99.63	98.34	0.34
**Pre-trained CNN (DenseNet 201) + PSO**									
Decision Tree	97.82	94.84	98.14	1.86	5.16	92.63	99.57	93.72	2.18
SVM	99.37	99.09	99.40	0.60	0.91	96.25	99.91	97.65	0.63
KNN	98.75	99.01	98.73	1.27	0.99	94.61	99.91	96.76	1.25
Ensemble	98.91	98.46	98.81	1.19	1.54	96.84	98.91	97.64	1.09
Naïve Bayes	99.06	97.71	99.57	0.43	2.29	95.68	99.40	96.68	0.94
Discriminant	98.75	99.01	98.73	1.27	0.99	97.19	99.91	98.09	1.25

**Table 4:** On a brain tumor dataset, many pre-trained CNNs were used with the PSO feature selection approach.

Pre-Trained CNN with PSO (Brain Tumor Dataset)									
Classifiers	ACC	TPR	TNR	FPR	FNR	PPV	NPV	F1-SCORE	MR
**Pre-trained CNN (AlexNet) + PSO**									
Decision Tree	89.39	82.70	92.28	7.72	17.30	82.25	92.50	82.47	10.61
SVM	97.87	96.25	98.59	1.41	3.75	96.77	98.36	96.51	2.13
KNN	98.69	97.34	99.29	0.71	2.66	98.38	98.82	97.86	1.31
Ensemble	95.75	93.47	96.73	3.27	6.53	92.47	97.18	92.97	4.25
Naïve Bayes	88.09	86.93	96.33	3.67	13.07	92.47	92.98	89.61	11.91
Discriminant	97.55	95.72	98.35	1.65	4.28	96.23	98.12	95.97	2.45
**Pre-trained CNN (GoogleNet) + PSO**									
Decision Tree	87.76	81.00	90.55	9.45	19.00	77.95	92.03	79.45	12.24
SVM	96.41	93.15	97.87	2.13	6.85	95.16	96.95	94.14	3.59
KNN	93.80	87.23	97.32	2.68	12.77	94.08	93.67	90.53	6.20
Ensemble	92.98	85.92	96.37	3.63	14.08	91.93	93.44	88.82	7.02
Naïve Bayes	90.86	86.50	96.72	3.28	13.50	93.01	89.92	89.64	9.14
Discriminant	96.41	92.67	99.03	0.97	7.33	97.84	95.78	95.18	3.59
**Pre-trained CNN (ResNet 50) + PSO**									
Decision Tree	90.21	85.39	92.18	7.82	14.61	81.75	93.91	83.53	9.79
SVM	97.87	96.75	98.36	1.64	3.25	96.23	98.59	96.49	2.13
KNN	**98.85**	98.90	98.83	1.17	1.10	97.31	99.53	98.10	1.15
Ensemble	96.73	95.60	97.21	2.79	4.40	93.54	98.12	94.56	3.27
Naïve Bayes	90.51	88.72	97.95	2.05	11.28	95.69	91.08	92.07	9.49
Discriminant	97.22	94.96	99.27	0.73	5.04	98.38	97.42	96.64	2.78
**Pre-trained CNN (DenseNet 201) + PSO**									
Decision Tree	93.47	88.42	95.74	4.26	11.58	90.32	94.84	89.36	6.53
SVM	96.90	96.13	97.22	2.78	3.87	93.54	98.36	94.82	3.10
KNN	97.71	95.74	98.58	1.42	4.26	96.77	98.12	96.25	2.29
Ensemble	96.73	94.62	97.65	2.35	5.38	94.62	97.65	94.62	3.27
Naïve Bayes	93.31	95.49	96.84	3.16	4.51	93.01	96.64	94.23	6.69
Discriminant	96.90	94.96	98.11	1.89	5.04	95.69	97.42	95.32	3.10

**Table 5:** Proposed method with other previous methods for COVID-19, and brain tumor datasets.

Ref.	Journal	Year	Methods	Datasets	Classes	Results
[19]	IEEE International Conference	2018	PSO + SVM	Brain Tumor	Benign, malignant	95.23%
[17]	Springer Link	2021	CNN + (GLCM), (GLRM), (LBP) + PSO	COVID-19	COVID-19, Pneumonia, normal	98.06%
[8]	Journal Pre-proof	2020	CNN	COVID-19	COVID-19, non-COVID-19	86.27%
[68]	Indonesian Journal of Electronics and Instrumentation Systems (IJEIS)	2018	GLCM + CNN	Brain Tumor	Meningioma, glioma, pituitary	82.00%
[69]	The Information Technology Management (ICCMIT’20)	2020	GLCM + VGG16 + Softmax	Brain Tumor	Meningioma, glioma, pituitary	96.50%
[70]	Scientific Reports—Computer Science	2018	Capsule networks (CapsNets) + Softmax	Brain Tumor	Meningioma, glioma, pituitary	86.56%
[71]	Springer Link	2020	Stacked autoencoder + Softmax	COVID-19	Positive, negative	94.70%
[72]	Scientific Reports—Computer Science	2021	CNN + Autoencoder + SVM	COVID-19 (Private)	COVID-19, normal	96.05%
	**Proposed Method**	**2021**	**CNN + PSO + SVM**	**COVID-19**	**Meningioma, glioma, pituitary**	**99.76%**
	**Proposed Method**	**2021**	**Autoencoder + PSO + kNN**	**Brain Tumor**	**COVID-19, pneumonia, normal**	**99.51%**

**Table 6:** Comparison table between PSO, ACO, and GA in terms of time and results for the brain tumor dataset.

Dataset	Combined Methods	PSO			ACO			GA		
		Classifiers	Acc.	Time (h)	Classifiers	Acc.	Time (h)	Classifiers	Acc.	Time (h)
**COVID-19**	Autoencoder	SVM	98.83%	1:15:00	SVM	98.68%	0:27:00	KNN	97.98%	1:00:00
	CNN (AlexNet)	Discriminant	99.68%	4:12:00	Discriminant	99.53%	1:17:00	KNN	98.60%	2:07:00
	CNN (GoogleNet)	Discriminant	98.83%	**1:00:00**	SVM	98.91%	**0:21:00**	Naïve Bayes	98.13%	**0:40:00**
	CNN (ResNet 50)	SVM	**99.76%**	2:05:00	SVM	**99.61%**	0:43:00	KNN	98.60%	1:05:00
	CNN (DenseNet 201)	SVM	99.37%	2:00:00	Naïve Bayes	99.14%	0:39:00	KNN	**98.75%**	1:04:00
**Brain Tumor**	Autoencoder	KNN	**99.51%**	0:23:00	KNN	**99.18%**	0:11:00	Ensemble	96.24%	0:16:00
	CNN (AlexNet)	KNN	98.69%	0:58:00	Discriminant	98.69%	0:12:00	Ensemble	94.61%	0:30:00
	CNN (GoogleNet)	Discriminant	96.41%	**0:15:00**	Discriminant	96.73%	**0:05:00**	KNN	93.96%	**0:09:00**
	CNN (ResNet 50)	KNN	98.85%	0:30:00	KNN	97.87%	0:11:00	Ensemble	**97.06%**	0:16:00
	CNN (DenseNet 201)	KNN	97.71%	0:27:00	SVM	98.20%	0:09:00	Ensemble	96.57%	0:18:00

## Data Availability

Both datasets are available in a publicly accessible repository; COVID-19: The data presented in this study are openly available in [Kaggle], reference number [59]. Brain Tumor: The data presented in this study are openly available in [FigShare] at [https://doi.org/10.6084/m9.figshare.1512427.v5], reference number [60].

## Abbreviations

**Parameter**	**Abbreviation**
Accuracy	ACC
Sensitivity, Recall, hit rate, True Positive Rate	TPR
Specificity, Selectivity, True Negative Rate	TNR
False positive rate	FPR
False negative rate	FNR
Precision, Positive Predictive Value	PPV
Negative Predictive Value	NPV
F1 Score	F1 Score
Misclassification rate	MR
